# Regional femoral head Hounsfield units mirror acetabular undercoverage in acetabular dysplasia

**DOI:** 10.1002/jeo2.70824

**Published:** 2026-06-23

**Authors:** Pierre Laboudie, Riccardo Sala, Mattia Blancato

**Affiliations:** ^1^ Clinique du Sport Bordeaux‐Mérignac Groupe Vivalto Mérignac France; ^2^ Department of Orthopaedics and Traumatology, ASST Lecco University of Milan Lecco Italy

**Keywords:** acetabular dysplasia, CT attenuation, femoral head coverage, hip, Hounsfield unit

## Abstract

**Purpose:**

Acetabular dysplasia is a three‐dimensional (3D) disorder characterized by regional femoral head undercoverage and altered joint loading. Percentage femoral head coverage (%FHC) enables regional assessment of acetabular coverage, but its relationship with femoral head computed tomography (CT) attenuation, expressed in Hounsfield units (HU), remains unclear. This study evaluated associations between 3D acetabular coverage and femoral head HU values in symptomatic dysplastic hips.

**Methods:**

This retrospective radiologic study included 40 patients with bilateral acetabular dysplastic morphology and complete bilateral CT imaging scheduled for periacetabular osteotomy between 2023 and 2026, resulting in 80 hips. Total and regional %FHC were calculated in the anterolateral (AL), anteromedial (AM), posteromedial (PM) and posterolateral (PL) regions. Lateral %FHC was defined as the mean of AL and PL coverage. Femoral head HU values were measured in 12 regions of interest on three axial slices across the same four quadrants, and lateral HU was defined as the mean of AL and PL values. Associations were assessed using age‐adjusted linear mixed‐effects models with a random intercept for participant.

**Results:**

Intraobserver and interobserver reliability were good to excellent. Age was independently associated with lower HU values across models. Lateral %FHC was positively associated with lateral HU (*β* = 10.68 HU per 1% increase; 95% confidence interval [CI], 3.76–17.60; *p* = 0.002). Regionally, %FHC_AL was associated with anterolateral femoral head Hounsfield unit value (UH_AL; *β* = 10.59; 95% CI, 2.73–18.44; *p* = 0.009), and %FHC_PL was associated with posterolateral femoral head Hounsfield unit value (UH_PL; *β* = 7.36; 95% CI, 1.58–13.13; *p *= 0.013). Total, AM and PM coverage were not associated with corresponding HU values.

**Conclusion:**

In this exploratory imaging study, lower lateral, AL and PL 3D acetabular coverage were associated with lower HU values in corresponding femoral head regions. These findings suggest that regional femoral head HU mapping may provide an imaging correlate of spatial acetabular undercoverage in dysplastic hips, although its clinical, diagnostic and prognostic utility requires further validation.

**Level of Evidence:**

Level III, diagnostic study.

**Abbreviations**
AAVH.C: acetabular anteversion at the femoral head centre
AI: acetabular index
AL: anterolateral
AM: anteromedial
AP: anteroposterior
BMI: body mass index
CI: confidence interval
CT: computed tomography
HU: Hounsfield unit
ICC: intraclass correlation coefficient
IQR: interquartile range
LCEA: lateral centre‐edge angle
LMM: linear mixed‐effects model
PAO: periacetabular osteotomy
PL: posterolateral
PM: posteromedial
ROI: region of interest
SD: standard deviation
UH_AL: anterolateral femoral head Hounsfield unit value
UH_AM: anteromedial femoral head Hounsfield unit value
UH_Lat: lateral composite femoral head Hounsfield unit value
UH_PL: posterolateral femoral head Hounsfield unit value
UH_PM: posteromedial femoral head Hounsfield unit value
%FHC: percentage femoral head coverage

## INTRODUCTION

Acetabular dysplasia is a well‐recognized structural cause of hip pain and premature osteoarthritis, resulting from acetabular undercoverage and abnormal joint loading [7, 22].

Conventional two‐dimensional (2D) radiographic parameters, such as the lateral centre‐edge angle (LCEA) and acetabular index (AI), remain central to diagnosis and surgical decision making, but they do not fully capture the three‐dimensional (3D) and region‐specific nature of acetabular deficiency [11, 30].

Indeed, acetabular dysplasia is now recognized as a complex 3D disorder with distinct patterns of undercoverage. Nepple et al. [27] demonstrated that dysplastic hips may exhibit anterolateral (AL), posterolateral (PL) or global deficiency rather than a single uniform pattern. The Ottawa group further refined this concept by classifying symptomatic acetabular dysplasia according to the predominant direction of instability and undercoverage—anterior, posterior or global/lateral—thereby underscoring the limitations of conventional 2D radiographic measurements in accurately characterizing the true morphology of dysplastic hips [3, 35].

Percentage of femoral head coverage (%FHC), defined as the proportion of the femoral head surface covered by the acetabulum, may provide a more precise assessment of undercoverage, including whether the deficiency is predominantly AL or PL [8]. %FHC has also been associated with the natural history of acetabular dysplasia and a strong correlation exists between %FHC and postoperative outcomes after hip preservation surgery [1, 6, 10, 13, 14].

In parallel, computed tomography (CT)‐based bone density measurements may provide information on the long‐term mechanical environment of a joint. CT‐osteoabsorptiometry studies have shown that subchondral mineralization patterns reflect cumulative loading history, and regional bone density distribution may therefore be interpreted as a marker of chronic joint stress rather than only static morphology [15, 25]. Related work in acetabular fractures has also suggested that femoral head HU values may be associated with acetabular pathology and outcomes [32]. In acetabular dysplasia, this concept is clinically relevant because regional undercoverage may alter joint loading, contribute to instability‐related symptoms and influence surgical planning. However, whether spatial acetabular undercoverage is associated with corresponding regional femoral head HU patterns has not been established. Demonstrating such a relationship would not by itself establish clinical utility, but it would provide an imaging‐based rationale for future studies linking HU mapping to symptoms, instability phenotype, cartilage status and postoperative outcomes.

The purpose of this exploratory imaging study was to evaluate the association between acetabular coverage, quantified as %FHC and femoral head CT attenuation, expressed in Hounsfield units (HU), globally, laterally and regionally (AL, anteromedial [AM], PL and posteromedial [PM]). We hypothesized that lower lateral and regional %FHC would be associated with lower HU values in corresponding femoral head regions. Secondary aims were to assess associations between HU measures and conventional radiographic parameters used to characterize dysplasia, including acetabular version, femoral version, LCEA, AI and presence of acetabular retroversion, and to explore whether HU mapping provides imaging information beyond conventional 2D measurements.

## MATERIALS AND METHODS

### Study design

This was a retrospective radiologic study of a consecutive surgical cohort from a high‐volume private orthopaedic referral centre. The study was approved by the institutional review board (CERC‐VS‐2025‐10‐3).

### Cohort

This study analysed a consecutive cohort of patients scheduled for periacetabular osteotomy (PAO) between 2023 and 2026 at a high‐volume private hip preservation centre.

**Figure 1:**
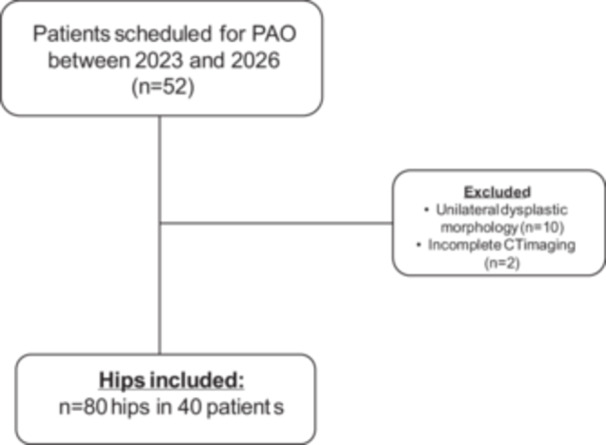
Flowchart of the patients. CT, computed tomography; PAO, periacetabular osteotomy.

Of 52 patients scheduled for PAO during the study period, only those with bilateral dysplastic morphology and complete bilateral CT imaging were included. Moreover, 12 patients were excluded, including 10 with unilateral morphologic involvement only and two with incomplete CT imaging, leaving 40 patients (80 hips) for analysis (Figure 1). The definition of acetabular dysplasia and surgical indication was based on symptomatic acetabular dysplasia with hip instability [34] according to the Ottawa classification [35], and inclusion was therefore not limited to conventional radiographic thresholds. The cohort included globally dysplastic hips (e.g., LCEA <25° with AI >10°) as well as anterior‐ or posterior‐predominant dysplasia identified on 3D CT assessment, with the direction of undercoverage determined primarily from 3D morphology rather than radiographs alone. Demographic and imaging characteristics of the patients are summarized in Table 1.

**Table 1:** Demographic and imaging characteristics of the study cohort.

Variable	Overall cohort
Patients and hips	
Patients, *n*	40
Hips, *n*	80
Female sex, *n* (%)	37 (92.5%)
Age, years, mean ± SD	28.26 ± 11.11 years
Body mass index, kg/m^2^, mean ± SD	22.46 ± 0.89
Radiographic parameters	
Lateral centre‐edge angle, °, mean ± SD	18.59 ± 4.70
Acetabular index, °, mean ± SD	8.74 ± 5.24
Acetabular retroversion (three positive radiographic signs), *n* (%)	33 (41.3%)
CT‐based alignment parameters	
Acetabular anteversion at femoral head centre, °, mean ± SD	21.64 ± 5.55
Femoral version, °, mean ± SD	22.25 ± 11.29

Abbreviations: CT, computed tomography; SD, standard deviation.

### Radiographic measurements

Conventional radiographic parameters were measured on a standardized supine anteroposterior (AP) pelvis radiograph. LCEA was defined as the angle between a vertical line through the femoral head centre and a line from the femoral head centre to the lateral edge of the sourcil, whereas AI was measured between the horizontal teardrop line and the sourcil line [5]. Acetabular retroversion was assessed using the crossover sign, posterior wall sign and ischial spine sign, and was considered present when all three signs were positive [21].

All patients underwent CT according to a standardized preoperative planning protocol used for the MyPAO process (Medacta). Acquisition consisted of a spiral axial scan including the entire pelvis and femur, with a maximum slice thickness of 1.0 mm. Automated 3D segmentation and reconstruction of the pelvis, femurs and sacrum were performed using Mimics 16.0 (Materialise), with manual correction when necessary.

Femoral version was measured on CT according to the method described by Murphy et al. [26]. Acetabular anteversion at the femoral head centre was measured on the corresponding axial slice as the angle between the line connecting the anterior and posterior acetabular rims and a posterior pelvic reference line.

**Figure 2:**
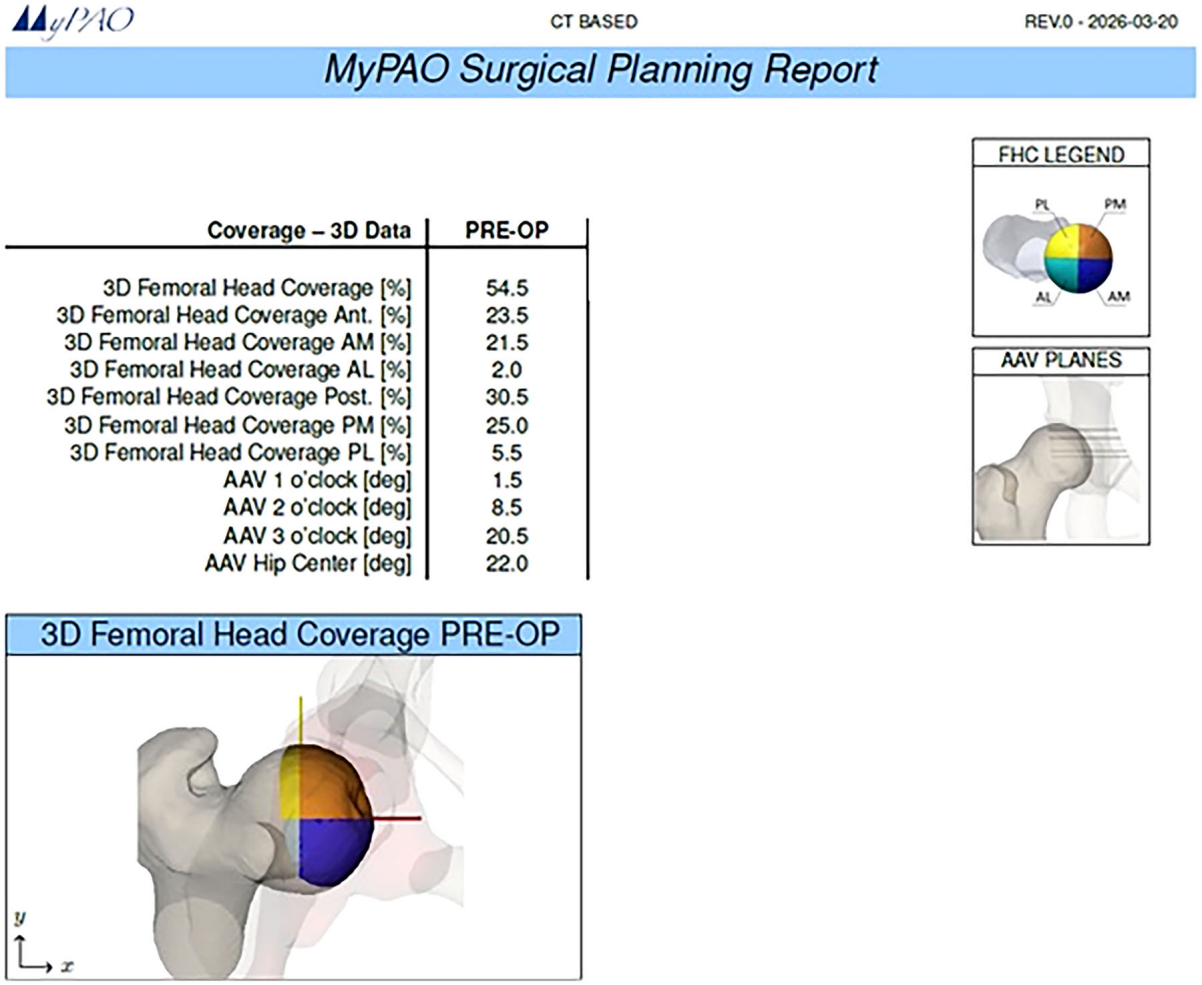
Representative MyPAO preoperative planning report (Medacta) illustrating 3D femoral head coverage and acetabular anteversion measurements. 3D, three‐dimensional; AAV, acetabular anteversion; AL, anterolateral; AM, anteromedial; FHC, femoral head coverage; PL, posterolateral; PM, posteromedial.

For the %FHC analysis, the pelvis was referenced to the anterior pelvic plane, defined by the bilateral anterior superior iliac spines and pubic tubercles. From these landmarks, an orthogonal pelvic coordinate system was generated to define the coronal, sagittal and axial pelvic planes. 3D %FHC was then calculated from the cranio‐caudal projection onto the axial pelvic plane. Total %FHC represented overall FHC, whereas regional coverage was subdivided into AL, PL, PM and AM quadrants. A composite lateral %FHC was defined as the mean of AL and PL coverage (Figure 2).

#### HU sampling and reliability

**Figure 3:**
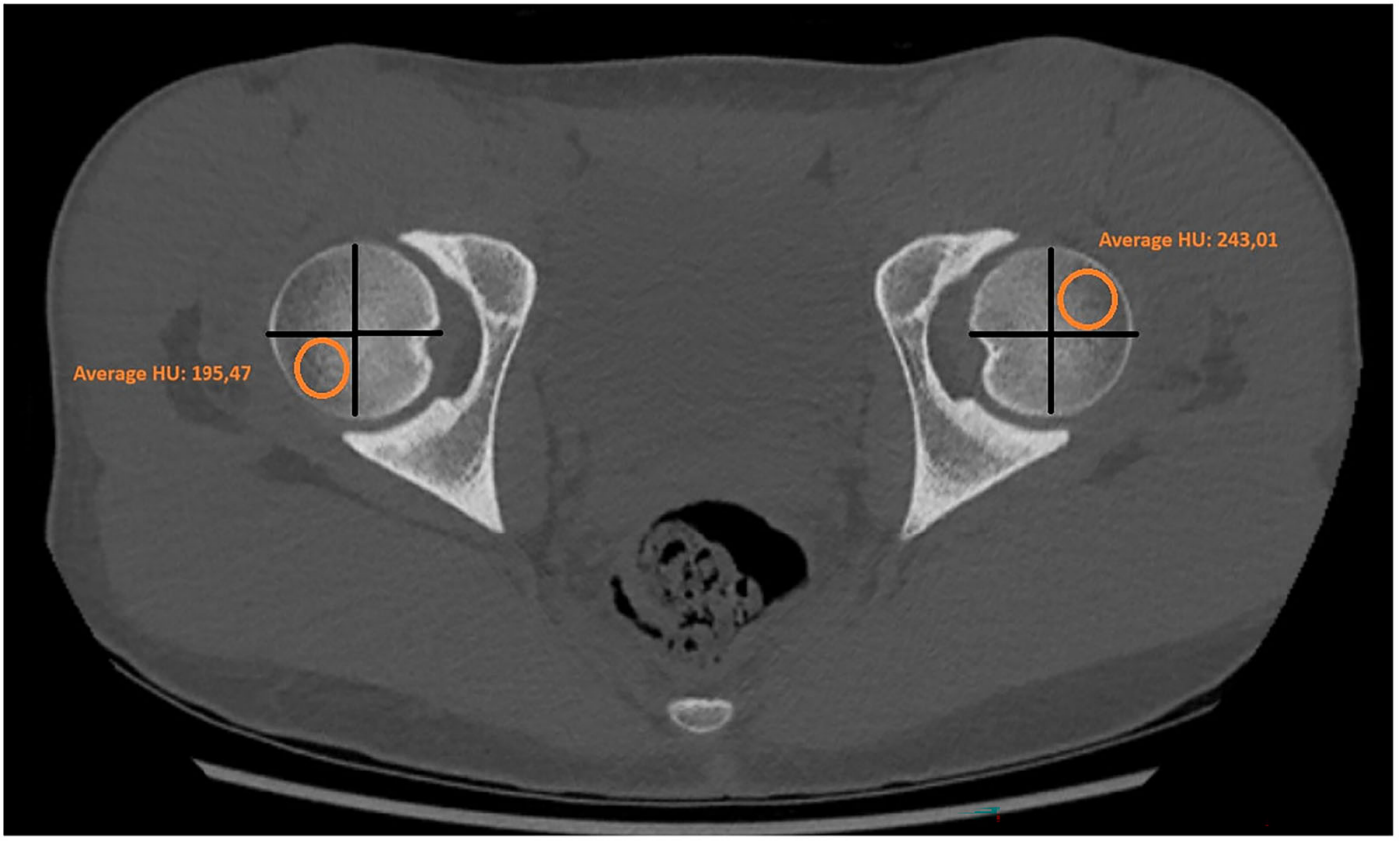
Quantification of femoral head HU after division of the femoral head into four quadrants. Shown are UH_PL measured in the right femoral head and UH_AL measured in the left femoral head. HU, Hounsfield unit; UH_AL, anterolateral femoral head Hounsfield unit value; UH_PL, posterolateral femoral head Hounsfield unit value.

On axial CT images, femoral head attenuation (HU) was assessed in the proximal weight‐bearing portion of the femoral head, above the physeal scar. To standardize sampling across hips, measurements were obtained on three consecutive axial slices (superior, middle and inferior) acquired at 1‐mm intervals, beginning at the fifth slice distal to the most superior aspect of the femoral head. This sampling strategy was defined a priori to avoid the extreme superior subchondral margin, where partial‐volume effects may reduce measurement reliability, while maintaining evaluation within the proximal femoral head expected to reflect chronic loading. Each slice was subdivided into four predefined quadrants—AL, AM, PM and PL—yielding 12 regions of interest (ROIs) per hip (3 slices × 4 quadrants). This quadrant‐based approach was selected to provide standardized regional sampling and to allow spatial comparison between local femoral head attenuation and corresponding patterns of acetabular coverage (Figure 3).

To assess measurement reliability, two independent observers each performed two separate HU measurements in all 12 ROIs. Intraobserver reliability was assessed separately for each observer using the two repeated measurements, whereas interobserver reliability was assessed by comparing the first measurement obtained by each observer. Reliability was quantified for each ROI using intraclass correlation coefficients (ICCs) with 95% confidence intervals (CIs).

For the final analysis, slice‐level HU values were aggregated into quadrant‐level variables to reduce the influence of local measurement variability and to provide a more robust estimate of regional femoral head attenuation. For each observer, the superior, middle and inferior HU values within a given quadrant were averaged to generate observer‐specific mean values (UH_AL, anteromedial femoral head Hounsfield unit value [UH_AM], posteromedial femoral head Hounsfield unit value [UH_PM] and posterolateral femoral head Hounsfield unit value [UH_PL]). Final quadrant‐level HU values were then obtained by averaging the two observer‐specific means. In addition, a composite lateral HU variable (UH_Lat) was defined as the mean of the final UH_AL and UH_PL values to reflect the lateral femoral head region, which was considered the region most directly relevant to lateral undercoverage in dysplastic hips.

### Statistical analysis

Continuous variables are reported as mean ± standard deviation (SD) or median (interquartile range [IQR]), as appropriate, and categorical variables as counts and percentages. Because both hips from the same participant could be included, associations between HU outcomes and imaging parameters were assessed using linear mixed‐effects models with a random intercept for participant. Age was entered a priori as a covariate in all models because of its known association with CT‐derived HU values and bone mineral density [2, 16, 29]. Results are reported as age‐adjusted *β* coefficients with 95% CIs and two‐sided *p* values.

No formal a priori sample size calculation was performed because this was a retrospective exploratory imaging study based on a consecutive PAO cohort. Although 80 hips were analysed, the effective sample size was limited by the number of independent patients (*n* = 40), and results were therefore interpreted primarily according to effect estimates and 95% CIs.

Additional exploratory analyses assessed associations between regional HU values and conventional radiographic parameters, including LCEA, AI, femoral version, acetabular anteversion and presence of acetabular retroversion. Models were fitted using restricted maximum‐likelihood estimation. Statistical significance was set at *p* < 0.05. All analyses were performed using Statistical Package for the Social Sciences (SPSS; IBM Corp).

## RESULTS

A total of 80 hips from 40 patients were included.

### ICC/reliability

Across the 12 zones, intraobserver reliability was good‐to‐excellent. For Reader 1, single‐measure ICCs ranged from 0.603 to 0.968 (median 0.935; IQR 0.913–0.954). For Reader 2, single‐measure ICCs ranged from 0.655 to 0.927 (median 0.867; IQR 0.812–0.891). Interobserver single‐measure ICCs ranged from 0.612 to 0.800 (median 0.738; IQR 0.665–0.768). Detailed ICCs by zone are reported in Table 2.

**Table 2:** Intraobserver and interobserver ICCs by zone.

Slice level	Quadrant	Zone ID	Intra ICC Reader 1 (95% CI)	Intra ICC Reader 2 (95% CI)	Inter ICC (95% CI)
Superior	AL	S‐AL	0.925 (0.885–0.951)	0.693 (0.559–0.792)	0.724 (0.600–0.814)
Superior	AM	S‐AM	0.905 (0.856–0.938)	0.752 (0.638–0.833)	0.667 (0.524–0.773)
Superior	PM	S‐PM	0.935 (0.900–0.958)	0.655 (0.510–0.764)	0.612 (0.454–0.732)
Superior	PL	S‐PL	0.954 (0.930–0.971)	0.858 (0.787–0.907)	0.707 (0.578–0.802)
Middle	AL	M‐AL	0.752 (0.638–0.833)	0.833 (0.751–0.890)	0.778 (0.674–0.852)
Middle	AM	M‐AM	0.915 (0.871–0.945)	0.832 (0.750–0.889)	0.622 (0.468–0.740)
Middle	PM	M‐PM	0.603 (0.443–0.726)	0.884 (0.825–0.924)	0.752 (0.638–0.834)
Middle	PL	M‐PL	0.939 (0.906–0.960)	0.901 (0.849–0.935)	0.800 (0.704–0.867)
Inferior	AL	I‐AL	0.968 (0.951–0.980)	0.911 (0.864–0.942)	0.764 (0.655–0.842)
Inferior	AM	I‐AM	0.955 (0.930–0.971)	0.877 (0.815–0.920)	0.788 (0.688–0.859)
Inferior	PM	I‐PM	0.936 (0.903–0.959)	0.887 (0.829–0.926)	0.755 (0.642–0.836)
Inferior	PL	I‐PL	0.957 (0.934–0.972)	0.927 (0.888–0.953)	0.660 (0.516–0.768)

*Note*: Zones defined as 3 axial slices (superior/middle/inferior) × 4 quadrants (AL/AM/PM/PL), all proximal to the physeal scar. ICCs shown are single‐measure ICCs extracted from SPSS outputs (two‐way mixed model; consistency/Type C) with 95% CIs.

Abbreviations: AL, anterolateral; AM, anteromedial; CIs, confidence intervals; ICCs, intraclass correlation coefficients; PL, posterolateral; PM, posteromedial; SPSS, Statistical Package for the Social Sciences.

#### Derived regional HU values

Across models, age was independently associated with lower HU values (approximately −2.9 to −3.9 HU per year; all *p* < 0.001). Derived regional HU values are detailed in Table 3.

**Table 3:** Descriptive statistics of derived regional HU values (averaged across slice levels).

Region	Mean ± SD (HU)	Min–max (HU)
AL	254.31 ± 72.84	90.92–491.92
AM	342.09 ± 70.03	195.75–512.83
PM	322.59 ± 79.35	138.08–537.25
PL	244.30 ± 74.84	96.08–493.25
Lat (mean of AL and PL)	249.30 ± 68.86	93.50–492.58

Abbreviations: AL, anterolateral; AM, anteromedial; HU, Hounsfield unit; PL, posterolateral; PM, posteromedial; SD, standard deviation.

##### Analyses

###### Primary analyses

**Figure 4:**
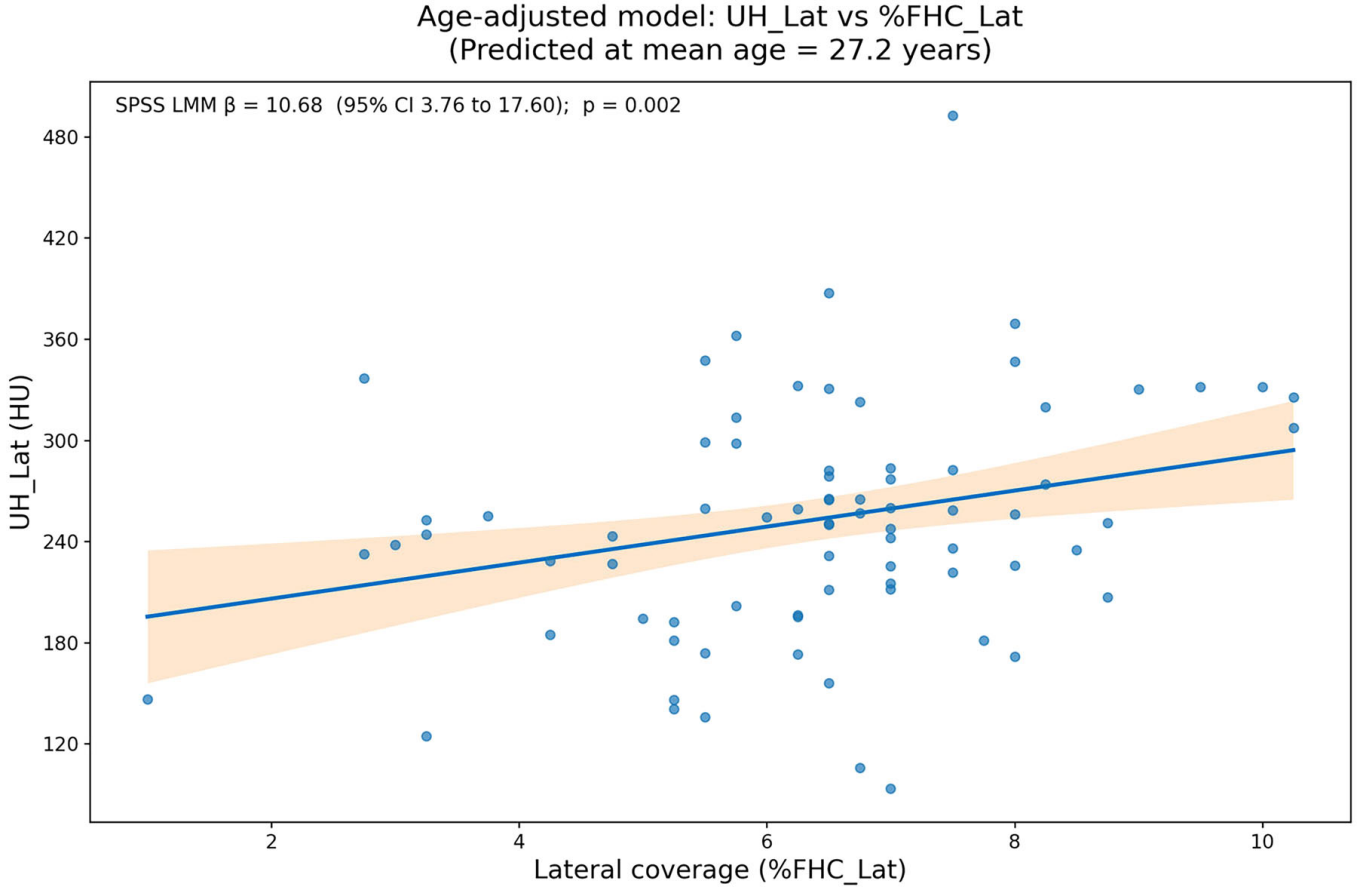
Age‐adjusted mixed‐effects model: UH_Lat versus %FHC_Lat with prediction line at mean age. Annotation reports SPSS LMM *β* and *p* value (age‐adjusted). CI, confidence interval; %FHC_Lat, percentage femoral head coverage lateral coverage; LMM, linear mixed‐effects model; SPSS, Statistical Package for the Social Sciences; UH_Lat, lateral composite femoral head Hounsfield unit value.

**Figure 5:**
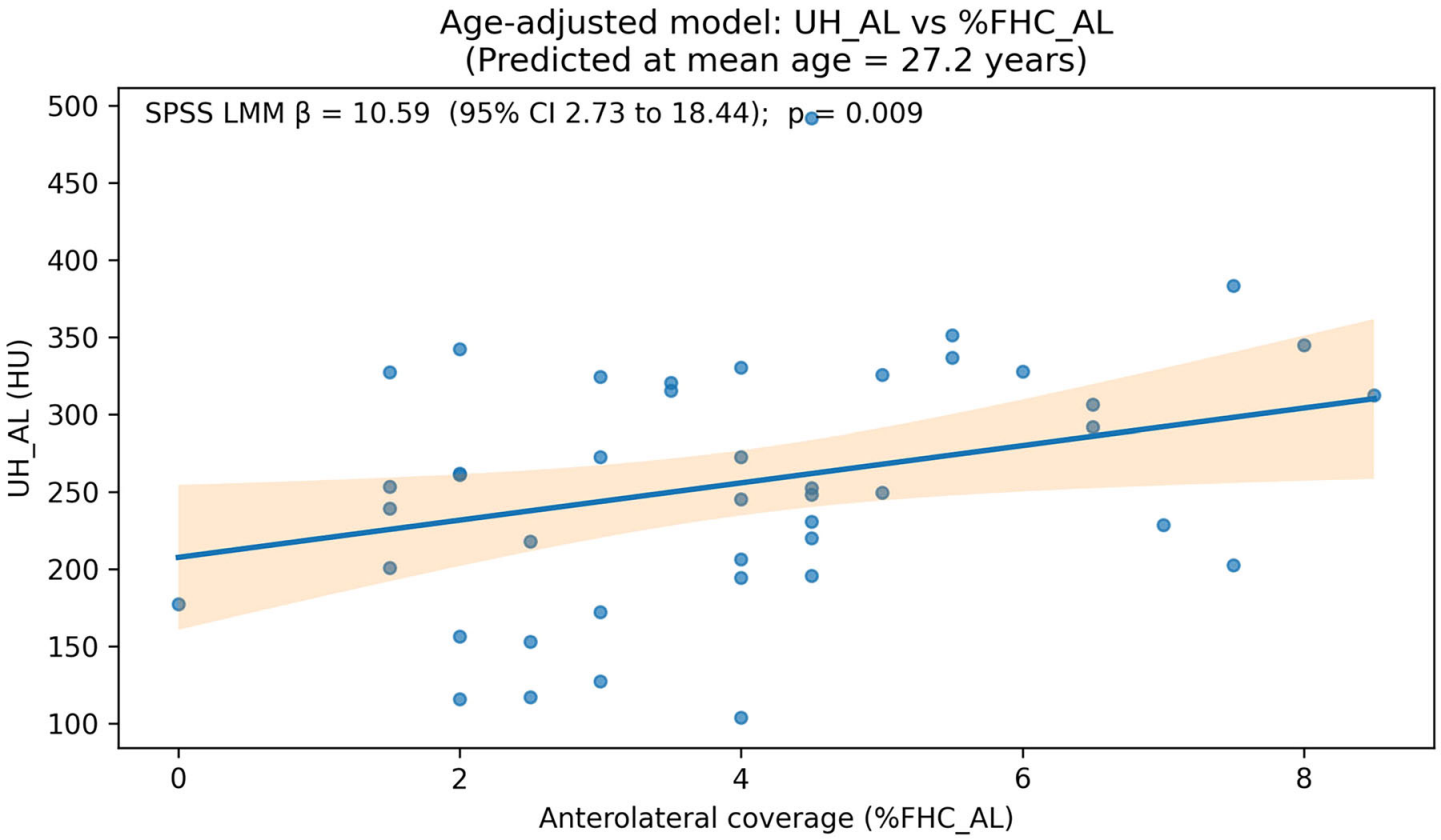
Age‐adjusted mixed‐effects model: UH_AL versus %FHC_AL with prediction line at mean age. Annotation reports SPSS LMM *β* and *p* value (age‐adjusted). CI, confidence interval; %FHC_AL, percentage femoral head coverage anterolateral coverage; LMM, linear mixed‐effects model; SPSS, Statistical Package for the Social Sciences; UH_AL, anterolateral femoral head Hounsfield unit value.

**Figure 6:**
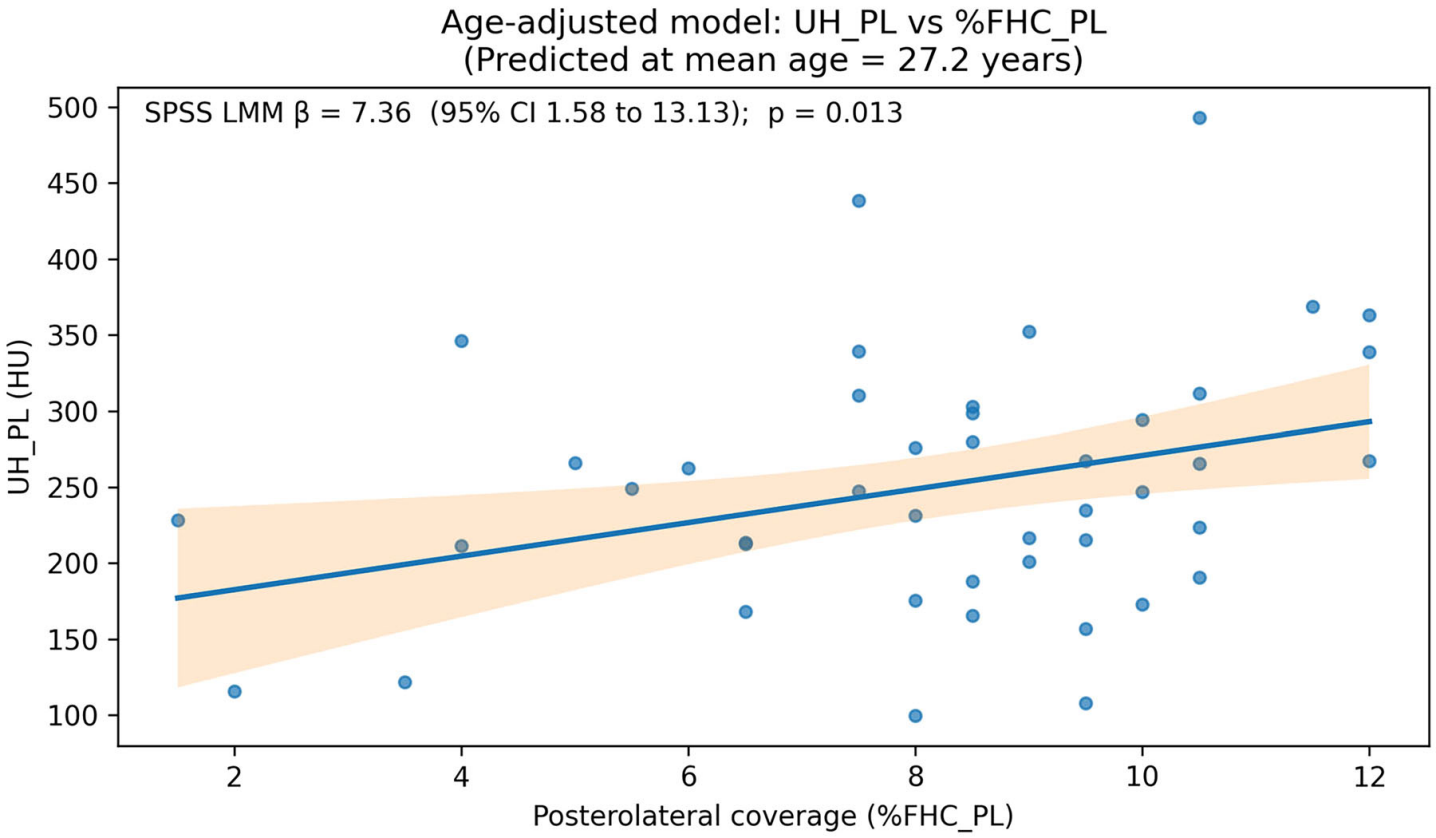
Age‐adjusted mixed‐effects model: UH_PL versus %FHC_PL with prediction line at mean age. Annotation reports SPSS LMM *β* and *p* value (age‐adjusted). CI, confidence interval; %FHC_PL, percentage femoral head coverage posterolateral coverage; LMM, linear mixed‐effects model; SPSS, Statistical Package for the Social Sciences; UH_PL, posterolateral femoral head Hounsfield unit value.

In age‐adjusted mixed‐effects models, lateral composite coverage (%FHC_Lat) was positively associated with UH_Lat (*β* = 10.68 HU per 1% increase; 95% CI, 3.76–17.60; *p* = 0.002; Figure 4). Likewise, AL coverage (%FHC_AL) remained positively associated with UH_AL (*β* = 10.59 HU per 1% increase; 95% CI, 2.73–18.44; *p* = 0.009; Figure 5). PL coverage (%FHC_PL) was also positively associated with UH_PL after age adjustment (*β* = 7.36; 95% CI, 1.58–13.13; *p* = 0.013; Figure 6). In contrast, total coverage was not significantly associated with total femoral head Hounsfield unit value (UH_total) after age adjustment (*β* = 2.11; 95% CI, −0.45 to 4.67; *p* = 0.105). No significant associations were observed for AM or PM regional analyses (Table 4).

**Table 4:** Primary analyses (age‐adjusted LMM with random intercept for patient).

Analysis	Dependent HU	Predictor	*β* (age‐adjusted)	95% CI	*p*
Global	UH_total	FHC_total	2.11	−0.45 to 4.67	0.105
AL	UH_AL	FHC_AL	10.59	2.73 to 18.44	0.009
PL	UH_PL	FHC_PL	7.36	1.58 to 13.13	0.013
AM	UH_AM	FHC_AM	0.41	−8.25 to 9.07	0.925
PM	UH_PM	FHC_PM	−24.29	−173.95 to 125.37	0.747
Lateral composite	UH_Lat	FHC_Lat	10.68	3.76 to 17.60	0.002

Abbreviations: AL, anterolateral; AM, anteromedial; CI, confidence interval; FHC_AL, anterolateral femoral head coverage; FHC_AM, anteromedial femoral head coverage; FHC_Lat, lateral femoral head coverage; FHC_PL, posterolateral femoral head coverage; FHC_PM, posteromedial femoral head coverage; FHC_total, total femoral head coverage; HU, Hounsfield unit; LMM, linear mixed‐effects model; PL, posterolateral; PM, posteromedial; UH_AL, anterolateral femoral head Hounsfield unit value; UH_AM, anteromedial femoral head Hounsfield unit value; UH_Lat, lateral composite femoral head Hounsfield unit value; UH_PL, posterolateral femoral head Hounsfield unit value; UH_PM, posteromedial femoral head Hounsfield unit value; UH_total, total femoral head Hounsfield unit value.

#### Secondary and exploratory analyses

**Figure 7:**
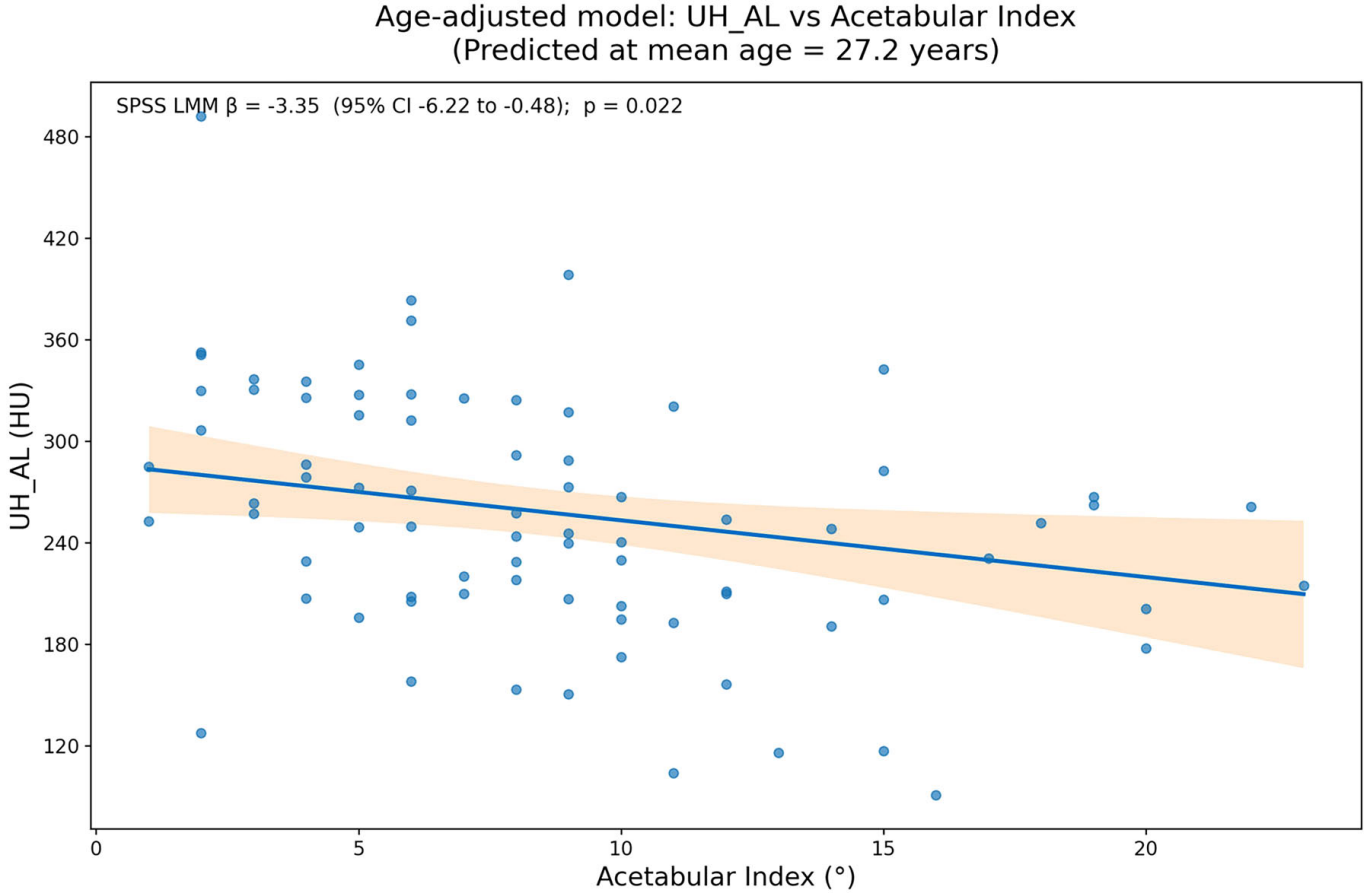
Age‐adjusted mixed‐effects model: UH_AL versus acetabular index (°) with prediction line at mean age. Annotation reports SPSS LMM *β* and *p* value (age‐adjusted). CI, confidence interval; LMM, linear mixed‐effects model; SPSS, Statistical Package for the Social Sciences; UH_AL, anterolateral femoral head Hounsfield unit value.

In the secondary and exploratory analyses, LCEA, femoral version, acetabular anteversion and presence of acetabular retroversion were not significantly associated with regional femoral head HU values after age adjustment. AI was inversely associated with UH_AL (*β* = −3.35 HU per 1°; 95% CI, −6.22 to −0.48; *p* = 0.022; Figure 7), but not with UH_total, UH_PL or UH_Lat. Detailed results are presented in Table 5.

**Table 5:** Secondary and exploratory analyses using age‐adjusted LMM with random intercept for participant.

Dependent HU	Predictor	*β* (age‐adjusted)	95% CI	*p*
UH_total	LCEA (°)	−0.41	−2.95 to 2.12	0.747
UH_Lat	LCEA (°)	0.32	−2.43 to 3.08	0.816
UH_AL	LCEA (°)	2.40	−0.53 to 5.33	0.107
UH_PL	LCEA (°)	−1.76	−4.83 to 1.32	0.259
UH_total	Femoral version (Murphy°)	0.84	−0.63 to 2.31	0.255
UH_Lat	Femoral version (Murphy°)	0.07	−1.57 to 1.72	0.929
UH_AL	Femoral version (Murphy°)	−0.51	−2.28 to 1.27	0.569
UH_PL	Femoral version (Murphy°)	0.65	−1.15 to 2.45	0.469
UH_total	Acetabular index (°)	−0.90	−3.36 to 1.55	0.466
UH_Lat	Acetabular index (°)	−2.10	−4.73 to 0.54	0.117
UH_AL	Acetabular index (°)	−3.35	−6.22 to −0.48	0.022
UH_PL	Acetabular index (°)	−1.24	−4.24 to 1.76	0.413
UH_Lat	AAVH.C (°)	−1.12	−3.72 to 1.47	0.390
UH_AL	AAVH.C (°)	−2.18	−4.94 to 0.58	0.120
UH_PL	AAVH.C (°)	−0.07	−2.96 to 2.82	0.963
UH_AL	Presence of acetabular retroversion	19.20	−13.66 to 52.06	0.247
UH_PL	Presence of acetabular retroversion	2.56	−31.91 to 37.03	0.883
UH_Lat	Presence of acetabular retroversion	10.13	−20.40 to 40.65	0.510

*Note*: *β* values for acetabular retroversion represent retroversion present versus absent and were obtained from age‐adjusted LMM with a random intercept for participant.

Abbreviations: AAVH.C, acetabular anteversion at the femoral head centre; AL, anterolateral; CI, confidence interval; HU, Hounsfield unit; Lat, lateral composite; LCEA, lateral centre‐edge angle; LMM, linear mixed‐effects model; PL, posterolateral; UH, femoral head attenuation; UH_AL, anterolateral femoral head Hounsfield unit value; UH_Lat, lateral composite femoral head Hounsfield unit value; UH_PL, posterolateral femoral head Hounsfield unit value; UH_total, total femoral head Hounsfield unit value.

**Figure 8:**
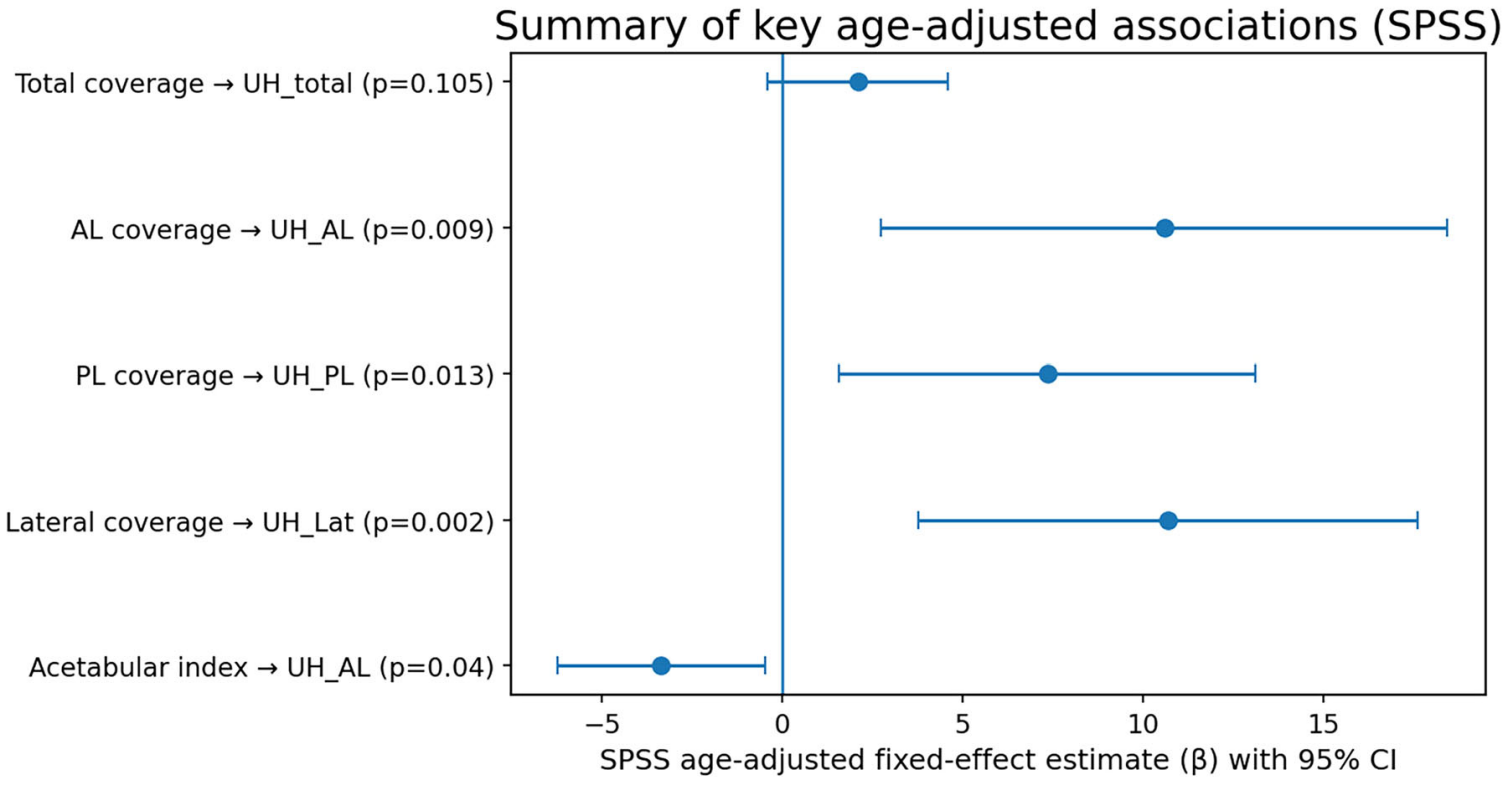
Forest plot summarizing key age‐adjusted associations using SPSS *β* estimates with 95% CIs (*p* values displayed in labels). AL, anterolateral; CI, confidence interval; PL, posterolateral; SPSS, Statistical Package for the Social Sciences; UH_AL, anterolateral femoral head Hounsfield unit value; UH_Lat, lateral composite femoral head Hounsfield unit value; UH_PL, posterolateral femoral head Hounsfield unit value; UH_total, total femoral head Hounsfield unit value.

The key age‐adjusted associations between radiographic parameters and HU values are summarized in a forest (Figure 8).

## DISCUSSION

The principal finding of this exploratory imaging study was a spatial association between regional acetabular undercoverage and femoral head CT attenuation in acetabular dysplasia. Specifically, lower lateral and regional AL and PL %FHC values were associated with lower HU values in the corresponding femoral head regions. This finding suggests that acetabular dysplasia may be associated with region‐specific differences in chronic femoral head loading rather than a uniform alteration of joint mechanics. The biologic plausibility of this observation is supported by the broader CT‐osteoabsorptiometry literature, which has shown across several joints that subchondral or juxta‐articular CT density distribution reflects cumulative mechanical loading over time and can therefore be interpreted as a marker of long‐term joint function rather than a purely static imaging parameter [15, 18, 25].

In this context, %FHC may reflect not only acetabular coverage but also the mechanical environment of the dysplastic hip. To our knowledge, this is the first study to report a direct spatial relationship between 3D FHC and regional femoral head HU distribution in acetabular dysplasia. The pattern observed is consistent with the concept of structural instability, but this interpretation remains inferential. Prior biomechanical and finite element studies have shown that reduced acetabular coverage alters joint contact mechanics by decreasing contact area and redistributing contact stresses within the hip joint [4, 33, 37, 38]. Dynamic analyses have also shown abnormal femoral head motion in dysplastic hips, and prior work on hip microinstability has described instability‐related imaging findings [9, 12, 19, 24, 28, 31, 36]. Accordingly, regional hypodensity of the femoral head may represent an imaging correlate of altered long‐term loading in the direction of undercoverage. However, the present study does not demonstrate dynamic instability directly and cannot establish a diagnostic or prognostic role for HU mapping.

Another important finding is that the primary associations were observed with %FHC, a 3D regional coverage metric, whereas conventional 2D radiographic parameters such as LCEA were not associated with regional HU values in this cohort. This observation suggests that regional %FHC may capture spatial information not reflected by planar radiographic measurements alone. This interpretation is consistent with the work of Nepple et al. [27], who demonstrated that dysplastic hips exhibit distinct 3D deficiency patterns rather than a single uniform morphology, and with the Ottawa classification, which categorizes acetabular dysplasia as global, anterior or posterior based on the predominant pattern of undercoverage [3, 35]. Prior CT‐based work has also shown that quantitative 3D coverage assessment may provide more detailed and reproducible characterization of acetabular morphology than isolated planar measurements [8, 19, 20], while postoperative outcomes after hip preservation surgery have been associated with 3D coverage parameters [13, 14].

Our results therefore provide an imaging correlate to these morphologic concepts by showing that regional acetabular deficiency is associated with regionally matched femoral head HU distribution. The exploratory analysis based on acetabular retroversion did not show an independent association between radiographic retroversion and regional femoral head HU values after age adjustment, suggesting that simple radiographic signs of retroversion may not capture the same regional loading information as 3D %FHC.

## FUTURE DIRECTIONS

Because no patient‐reported outcomes, symptom‐severity measures, cartilage assessment or postoperative follow‐up were analysed, the present findings should not be interpreted as evidence that HU mapping improves diagnosis, prognosis or surgical decision‐making. Rather, they should be considered hypothesis‐generating and provide a rationale for future clinically oriented studies.

First, these findings support further evaluation of CT‐based 3D assessment in acetabular dysplasia, including quantitative coverage metrics such as %FHC. In the present study, regional %FHC was associated with spatially corresponding femoral head HU values, whereas conventional 2D radiographic parameters were not. This suggests that 3D coverage analysis may capture biomechanically relevant information beyond conventional radiographs and may also be integrated into dynamic analyses [23]. Accordingly, it may be useful for future refinements in the evaluation and preoperative planning of dysplastic hips.

Second, regional femoral head HU mapping may represent a complementary imaging marker in selected patients with suspected hip instability, particularly in borderline or complex morphologic presentations. In such cases, reduced regional femoral head HU values could reflect chronic unloading related to subtle or dynamic instability, providing information that is not captured by static radiographic parameters alone and potentially helping to explain the imperfect correlation between patient‐reported outcome measures and the radiographic severity of acetabular dysplasia [17]. However, this hypothesis requires validation against clinical symptoms, dynamic instability assessment, cartilage status and patient‐reported outcomes.

Third, integrating regional HU mapping into 3D preoperative planning may help bridge static morphology and the time‐dependent mechanical consequences of hip instability. Current radiographic and CT measurements describe morphology at a single time point, whereas regional HU distribution may reflect the cumulative effects of loading over months or years. Future work combining 3D morphologic analysis, dynamic instability assessment and regional bone density mapping may therefore improve understanding of how dysplastic hips function over time and may help determine whether specific regions should be preferentially addressed during joint‐preserving surgery [12, 19, 24, 28, 36].

Finally, future prospective studies should determine whether regional HU patterns are associated with symptom severity, patient‐reported outcomes, dysplasia subtype, cartilage damage, dynamic instability or postoperative improvement after PAO. The development of clinically meaningful thresholds or cut‐offs would require larger cohorts with external validation and longitudinal follow‐up. Such studies will be necessary before HU mapping can be considered a clinically actionable tool in the evaluation or treatment planning of acetabular dysplasia.

## LIMITATIONS AND STRENGTHS

This study has several limitations. Its retrospective design precludes causal inference. The sample size was modest and was not based on an a priori power calculation, as this was an exploratory study of a consecutive imaging cohort. Although 80 hips were analysed, they were obtained from 40 independent patients, which limits statistical power, particularly for secondary and regional analyses. Therefore, non‐significant findings should be interpreted cautiously, as they may reflect limited precision and possible Type II error rather than definitive absence of association. The cohort consisted of symptomatic patients selected for PAO, which may limit generalizability. In addition, CT attenuation is influenced by systemic factors such as age and bone quality, although age was accounted for in all analyses. Another important limitation is the absence of clinical outcome data, including patient‐reported outcome measures, symptom severity, cartilage status and postoperative follow‐up after PAO. Therefore, the present study cannot determine whether regional HU values have diagnostic, prognostic or treatment‐guiding value. Finally, the HU sampling protocol was defined a priori but remains arbitrary, as no established method exists for regional femoral head HU assessment in dysplastic hips.

Despite these limitations, this study has several strengths. It incorporates bilateral data with appropriate mixed‐effects modelling, allowing statistical handling of within‐patient correlations. The use of a standardized 3D coverage assessment and a predefined regional HU mapping protocol enhances reproducibility. Furthermore, the good‐to‐excellent intraobserver and interobserver reliability supports the robustness of the measurement methodology. All analyses were age‐adjusted, and the regional approach allowed direct spatial comparison between acetabular coverage and femoral head CT attenuation. To our knowledge, this is the first study to demonstrate a spatial correspondence between acetabular undercoverage and femoral head HU distribution in acetabular dysplasia.

## CONCLUSION

In this exploratory radiologic study, lower lateral, AL and PL 3D acetabular coverage were associated with lower HU values in the corresponding femoral head regions. These findings support a spatial relationship between acetabular undercoverage and femoral head CT attenuation in symptomatic dysplastic hips. Importantly, regional HU values were associated with 3D %FHC but not with conventional 2D radiographic parameters, supporting the relevance of 3D characterization of acetabular dysplasia. However, HU mapping should currently be considered an exploratory imaging marker, and its diagnostic, prognostic and treatment‐planning utility requires prospective clinical validation.

## AUTHOR CONTRIBUTIONS

Pierre Laboudie conceived and designed the study, supervised data collection, interpreted the results and drafted the manuscript. Riccardo Sala and Mattia Blancato contributed to imaging measurements, data collection and data interpretation. All authors critically revised the manuscript for important intellectual content, approved the final version and agree to be accountable for all aspects of the work.

## FUNDING INFORMATION

The authors have no funding to report.

## CONFLICT OF INTEREST STATEMENT

Pierre Laboudie is a consultant for Medacta, Stryker and Amplitude. The remaining authors declare no conflict of interest.

## ETHICS STATEMENT

Ethical approval for this study was obtained from Vivalto Santé, CERC‐VS‐2025‐10‐3. Written informed consent was obtained from all participants included in the study.

## Data Availability

Anonymized data may be available from the corresponding author upon reasonable request and subject to institutional approval.
